# An analysis of online messages about probiotics

**DOI:** 10.1186/1471-230X-13-5

**Published:** 2013-01-11

**Authors:** Margaret A Brinich, Mary Beth Mercer, Richard R Sharp

**Affiliations:** 1 Department of Bioethics, Cleveland Clinic, 9500 Euclid, Ave., JJ60, Cleveland, OH, 44195, USA; 2 Center for Genetic Research Ethics & Law, Case Western Reserve University, Cleveland, OH, 44106, USA

**Keywords:** Probiotics, Doctor-patient communication, Complementary and alternative medicine, Ethics

## Abstract

Internet websites are a resource for patients seeking information about probiotics. We examined a sample of 71 websites presenting probiotic information. We found that descriptions of benefits far outnumbered descriptions of risks and commercial websites presented significantly fewer risks than noncommercial websites. The bias towards the presentation of therapeutic benefits in online content suggests that patients are likely interested in using probiotics and may have unrealistic expectations for therapeutic benefit. Gastroenterologists may find it useful to initiate conversations about probiotics within the context of a comprehensive health management plan and should seek to establish realistic therapeutic expectations with their patients.

## Findings

A recent analysis by the Pew Internet & American Life Project found that 80% of Internet users in the U.S. search the Internet to obtain health-related information [1]. Those who are living with chronic disease report going online to search for health related information at a slightly higher rate (83%) [1]. These trends reflect the increasing popularity of the Internet as a key source of information about health and highlight the need for physicians to be familiar with the content of health-related websites their patients may be accessing [2-5].

Like many patients with chronic illness, individuals with gastrointestinal (GI) diseases often turn to the Internet for information on treatment options [6]. Of particular interest to many patients living with inflammatory bowel disease (IBD), irritable bowel syndrome (IBS), and other chronic GI diseases is the potential utility of probiotics and other so-called complementary and alternative medicines [7]. Patients can purchase probiotics directly and the marketing of these products is not regulated by the Food and Drug Administration (FDA) in a manner that is comparable to the regulation of pharmaceutical drugs. These considerations highlight the need to examine Internet messages about probiotics. Knowing what information is communicated to patients, and by whom, is critical for gastroenterologists and other clinicians who care for patients with chronic GI diseases, many of whom are using probiotics or may be considering their use as a supplement to ongoing care regimens [7-10].

We present a systematic description of Internet websites discussing probiotics. Our analysis describes: *1-* Internet depictions of the potential benefits and risks of probiotics; *2-* common misrepresentations of probiotics; and, *3-* other information that may influence patient decisions about the use of probiotics. To the extent that these Internet messages may shape patient attitudes and beliefs, gastroenterologists should be aware of how the potential benefits and risks of probiotics are being presented to the patients they serve. By understanding the full spectrum of Internet messages concerning probiotics, gastroenterologists can better engage their patients in discussions about probiotics and their potential use as a tool for managing chronic GI diseases.

**Figure 1:**
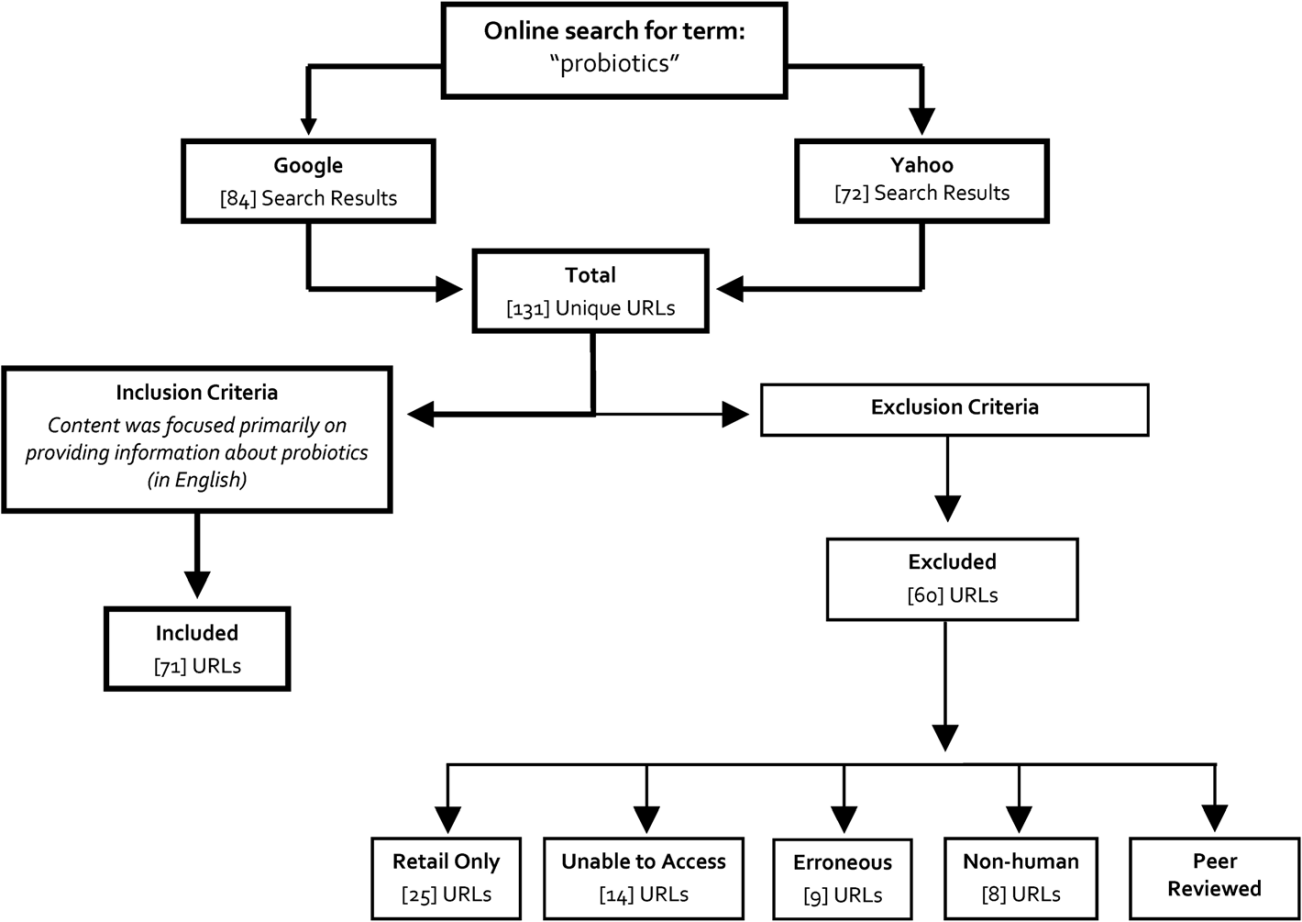
Description of Sampling Methods.

We constructed a sample of probiotic-related websites using criteria that would mirror the search patterns of patients with GI diseases. Online searches were completed initially using the five most popular search engines at the time of sampling—Google, Yahoo, LiveSearch, AOL, and Ask [11]. Due to the similar search algorithms employed by these five search engines, keyword searches using the term “probiotics” returned similar results. Therefore, we chose to rely on two popular search engines, Google and Yahoo, in identifying relevant Internet websites. We conducted Internet queries using these search engines from May 6 to 26, 2009. Based on the work of Eysenbach and colleagues, which suggests that consumers’ online health information searches are typically limited to initial search results, we limited our sample to the first 50 results of each search engine query (5 pages of results per query) [12]. Websites were excluded from the sample if the website: *1-* existed solely as a retail site intended for direct purchase; *2-* was not able to be accessed at the time of sample selection; *3-* was determined to be an erroneous result upon closer examination (e.g. a clearly unrelated website with no content related to probiotics); *4-* focused on non-human applications of probiotics; or *5-* was a peer reviewed scientific, medical, or research article or book (Figure 1).

Due to the highly dynamic nature of online content, discrete images of each website were saved as PDF files along with the date on which the content was captured. Two analysts independently reviewed each of these files using a code sheet to document specific content areas and qualities of the websites. A code sheet was developed and used to evaluate major content areas (Table 1). The Sandvik scale was used to evaluate overall website quality based on seven characteristics measured on a scale from 0-worst to 2-best [13]. These results were then summed for a best possible score of 14 [13].

**Table 1:** Coding categories used to analyze the content of 71 Internet websites on probiotics

**Content Domain**	**Research Question**
**Framework**	*How are probiotics being defined for the reader?*
**Mechanism of Action**	*By which biologic mechanisms do probiotics function within the body?*
**Product**	*What products are mentioned and how much detail is given about their contents?*
**Regulation**	*How is the regulatory status of probiotics described (if at all)?*
**Benefits**	*What benefits are attributed to using probiotics?*
**Risks**	*What risks are attributed to using probiotics?*
**Research**	*What research about probiotics is described (if any)?*
**References**	*What types of materials are cited (if any)?*
**Tone**	*Is there a clear recommendation for or against probiotic use? How is this conveyed?*
**Accessibility**	*In what ways is the website connected to additional information and probiotic products?*
**Website** **Characteristics**	*Describe the structure and functionality of the website itself.*
**Sandvik Scale**	*A 7-item instrument used to rate the overall quality of a website.*

After two analysts independently coded websites for content, their respective code sheets were compared and used to establish a single consensus code sheet. Cohen’s kappa statistic was used to evaluate agreement between the two analysts and showed very high levels of agreement across the 10153 items (143 items per website) evaluated [14]. Kappa values for each of the 71 websites ranged from 0.41 to 0.94, with a mean value of 0.72 and standard deviation of 0.10. Results were entered into an Access database and imported into SPSS version 19.0 for further analysis [15]. Descriptive statistics were examined to check for outliers and data-entry errors. Independent samples t-tests (for ordinal data) and Fisher’s exact tests (for nominal data) were used to assess differences between commercial and noncommercial websites. P values of < 0.05 were considered statistically significant.

A total of 71 websites were identified and analyzed for content. As shown in Table 2, these websites were diverse with respect to content and intended audience. Approximately half of the websites (51%) were categorized as commercial. Websites that were not commercial in nature included those run by non-profit organizations (13%), news media (13%), Internet content farms^a^ (4%), and personal websites (4%). Two websites included in the sample were maintained by medical institutions, one from the Mayo Clinic and another from the University of Alabama Health System. The only government site included in the analysis was supported by the National Center for Complementary and Alternative Medicine.

**Table 2:** Characteristics of 71 Internet websites on probiotics

**Characteristic**	**N *(%)***
**Type of Website**	
commercial	36 *(51)*
media	9 *(13)*
organization	9 *(13)*
other	9 *(13)*
content farms	3 *(4)*
personal	3 *(4)*
healthcare institution	2 *(3)*
government	1 *(1)*
**Information Provided**	
organization maintaining web address	67 *(94)*
year established	58 *(82)*
last updated	31 *(44)*
**External Links**	
commercial	46 *(65)*
research	21 *(30)*
educational	12 *(17)*
government	11 *(16)*
other	10 *(14)*
other organization	8 *(11)*
voluntary health organization	8 *(11)*
medical institution	6 *(9)*
professional organization	6 *(9)*
media	4 *(6)*
**Link to Products**	
at least 1 link to a probiotic product	51 *(72)*
advertisement	38 *(75)*
link to outside url to purchase	24 *(47)*
information on where to purchase	17 *(33)*
direct purchase	14 *(28)*
**Sponsorship**	
returned as sponsored link	12 *(17)*
**Contact Information**	
provides contact information	58 *(82)*

A majority of these websites (76%) identified at least one probiotic product using a commercial brand name. It was also common for websites to have some connection or “linkage” to more information about a specific probiotic product (72%) (Table 2). Many of those links were to product advertisements (75%). Other links directed readers to outside websites where probiotic products could be purchased (47%).

By utilizing a standard metric to examine website quality, the Sandvik score set a mean overall quality score at 9.3 (Table 3). When commercial and noncommercial websites were compared, their Sandvik scores differed significantly with mean scores of 7.7 and 10.7 respectively (p=.001). Commercial websites received consistently lower mean ratings than their noncommercial counterparts in six of the seven content areas examined using the Sandvik scale, including “balance,” “authorship,” and “currency” of content, with significantly lower mean scores across each of these three areas (p=.001).

**Table 3:** Comparison of general quality criteria scores (Sandvik Scores) between 71 commercial and noncommercial Internet websites

	$\;\bar{X}$		
	**Com**	**Non-Com**	**p-value**
**Ownership**	1.81	1.91	.319
**Source**	1.33	1.51	.336
**Interactivity**	1.33	1.23	.507
**Balance**	0.28	1.69	**.001**
**Authorship**	0.50	1.26	**.001**
**Currency**	0.94	1.60	**.001**
**Navigability**	1.58	1.69	.405
Total Score*	7.72	10.74	**.001**

* Each Sandvik criterion is scored on a scale of 0 to 2, for a total score of 0 (worst)-14 (best).

Websites were generally accurate in their depictions of what probiotics are and how they function in the host (Table 4). Probiotics typically were described as “complementary” to other approaches to promoting good health. Although a widely accepted definition of probiotics recommended by the Food and Agricultural Organization of the United Nations and World Health Organization (FAO-WHO) was not used consistently across websites, over half of the websites did contain all four components of this definition (56%). Two primary elements of this definition, that probiotics are “live microorganisms” (89%) ingested to provide “health benefit” (86%), were present in most websites. Websites often suggested that probiotics may provide benefits related to general health and wellness (70%) as well as improvements in GI health (96%).

**Table 4:** Characterizations of probiotics on 71 Internet websites

	**N *(%)***
Defined Using Elements of the FAO-WHO Definition*	
contain all 4 components of FAO-WHO definition	40 *(56)*
live microorganisms	63 *(89)*
administered to host	53 *(75)*
adequate amounts	42 *(59)*
health benefits	61 *(86)*
**Probiotics Function By**	
regulating local immune response	59 *(83)*
regulating inflammatory response	26 *(37)*
improving gut’s barrier function	26 *(37)*
inhibiting pathogenic bacteria colonization	58 *(82)*
other	28 *(39)*
**Indications for Use**	
improve general health	50 *(70)*
improve GI health	68 *(96)*
**Product**	
strain of bacteria noted	67 *(94)*
general vehicle for consumption noted	70 *(99)*
specific brand named	54 *(76)*

* The FAO-WHO defines probiotics as live microorganisms which when administered in adequate amounts confer a health benefit on the host (2002).

The description of probiotics as consumable products almost uniformly included some mention of the species (94%) and manner in which it is to be ingested (99%). In addition to defining what probiotics are and suggesting appropriate indications for their use, all but one website noted at least one mechanism of action by which probiotic bacteria function in the host. The two most common mechanisms described were to regulate immune response (83%) and inhibit pathogenic bacteria colonization (82%).

### Focus on therapeutic benefit

The representation of potential benefits and risks varied greatly across websites (Table 5). Seventy percent of websites identified at least one specific disease or clinical indication for probiotics. Specific benefits cited ranged from relieving general gastrointestinal distress such as gas and bloating (n=63), to managing behavioral symptoms related to autism (n=6) and preventing cardiovascular disease (n=4). Specific risks associated with probiotics were noted less frequently than benefits and typically focused on minor GI issues such as the potential for diarrhea or gas (n=28). A few websites also discussed less common risks related to bacterial infection (n=7) and headaches (n=4). Other potential safety issues focused on heightened risks for specific populations, such as probiotic use by children (n=20) and pregnant women (n=13). Of the websites examined, 47% did not mention any risks associated with probiotic use.

**Table 5:** Specific benefits and risks of probiotics described on 71 Internet websites*

**Benefits**	**Risks**
Allergies	GI (minor, e.g. wind, bloating)
Candida	Safety Issues for Children
Cholesterol Levels	Safety Issues for Immunocompromised
Colitis	Safety Issues for Pregnant
Diarrhea-*other*	Safety Issues for those with Underlying Health Issues
Eczema	
**GI (minor, e.g. wind, bloating)**^**†**^	
H-Pylori	
Hypertension	
**Improves Immune Function**^**†**^	
Improves Immune Absorption	
Infections	
Inflammatory Bowel Disease	
Irritable Bowel Syndrome	
Oral Health	
Prevent Cancer	
**Prevent Lactose Intolerance**^**†**^	
Reduces Antibiotic-Related Diarrhea	
Reduces Inflammation	
Reduces Toxins	
Respiratory Disease-*other*	
Rotavirus Diarrhea	
Skin Conditions-*other*	
Traveler’s Diarrhea	
Urinary Tract Infection	
Vaginal Health	
Vitamins	
Weight Management	

* These benefits and risks were cited by at least 10% of the 71 Internet websites examined. An additional 49 benefits and 22 risks were cited on these websites but not on at least 10% of the total sample and are not listed above.

† The benefits listed in bold appeared on more than 50% of the 71 Internet websites examined. No risks were described on more than 50% of the websites.

On average, websites mentioned 10.2 benefits and 1.8 risks per website, with an upper range of 36 benefits and 11 risks on any individual site (Table 6). The number of risks described on commercial websites was significantly lower than noncommercial websites (1.0 and 3.0 respectively; p=.002). Although no formal evaluation was performed, websites often reiterated one or more benefits of using probiotics multiple times throughout the text. Conversely, the discussion of risks associated with probiotics was less commonly reiterated in multiple locations.

**Table 6:** A comparison of how commercial and noncommercial websites portray probiotics

	**N *(%)***			
	***Total***	**Com**	**Non-Com**	**p-value**
**Stance**				
explicit endorsement of probiotic use	40 *(56)*	33 (83)	7 (17)	**.001**^**†**^
**Use of Probiotics Suggested by an Identifiable Individual**				
physician	16 *(23)*	10 (63)	6 (37)	.396
other medical professional	22 *(31)*	11 (50)	11 (50)	1.00
patient or consumer	15 *(21)*	10 (67)	5 (33)	.245
identified corporate representative	5 *(7)*	5 (100)	—	**.050**^**†**^
**Role of Physicians**				
encourage discussion with MD	33 *(47)*	16 (48)	17 (52)	.814
**Disclaimer**				
not FDA approved	14 *(20)*	12 (86)	2 (14)	**.006**^**†**^
“not intended as substitute for medical advice or care”	23*(32)*	15 (65)	8 (35)	.129
**General Statements of Benefit and Risk**				
statement indicating broad benefit of probiotics	42 *(59)*	26 *(62)*	16 *(38)*	**.031**^**†**^
statement indicating probiotics are generally safe	35 *(49)*	17 *(49)*	18 *(51)*	.814
**Number of Benefits and Risks Described**	$\;\bar{X}$	$\;\bar{X}$	$\;\bar{X}$	**p-value**
benefits	10.17 ± 8.27	10.58	9.74	.672
risks	1.79 ± 2.59	1.00	3.03	**.002**^**‡**^

† Fisher’s exact test.

‡ Student’s T-test.

Statements regarding the general safety of probiotics appeared in approximately half (49%) of the websites reviewed (Table 6). Broad statements of general health benefit—such as claims that probiotics can help *“Maintain optimal health and wellness”*—were present in more than half of the websites (59%). Commercial websites were significantly more likely to include such a statement (62%) in comparison to noncommercial websites (32%) (p=.031).

Nearly half (47%) of the websites encouraged readers to discuss probiotics with a heath care provider and 56% explicitly endorsed the use of probiotics (Table 6). These endorsements were often made by an identifiable individual, such as a named physician (23%), other medical professional (31%) or patient/consumer (21%). Commercial websites were more likely to recommend the use of probiotics than noncommercial websites (p=.001).

When gastroenterologists and other healthcare providers (HCPs) encounter patients who are interested in using probiotics as therapy for their GI disease, they should anticipate that many of these patients will have been exposed to a variety of messages concerning the health benefits of probiotics. Our data indicate that many Internet websites contain unsubstantiated and misleading claims about the therapeutic benefits of probiotics. This benefit-focused messaging is particularly troubling given that probiotics have not been studied extensively in clinical trials and there is little to no evidence supporting many of the benefits claimed on some websites. For example, although there is evidence suggesting that probiotic bacteria may have some utility in the care of patients with IBS, ulcerative colitis, and pouchitis [16-21], claims that probiotics can prevent cancer or assist in managing autistic behaviors lack empirical support.

In addition to unsupported claims of benefit, our analysis also revealed a comparative lack of information about the risks of using probiotics. Studies suggest that the level of risk posed to healthy individuals by a typical probiotic is very low [22]. In the absence of high quality data documenting harms associated with typical uses of probiotics, the lack of online information about possible side effects is unsurprising. For some individuals, however, such as immunocompromised patients who may be seeking probiotics to manage a chronic GI disease, there may be additional risks that are not discussed on most Internet websites [23-27].

### Effective patient communication amid mixed health messages

Alongside the specific health benefits noted above, our analysis identified many broad statements promoting probiotics as a general wellness tool. The promotion of probiotics as a holistic “complementary and alternative medicine” (CAM) situates these products in a market space occupied primarily by healthy consumers seeking to prevent disease and maintain overall health and wellness. Unfortunately, a number of studies have demonstrated that patients often are reluctant to discuss their interest in CAM with physicians [28-32]. In the case of probiotics, wellness messaging on Internet websites may make patients less likely to consult with a HCP prior to using probiotics. By employing a combination of marketing messages, focusing on both specific symptom management and promotion of overall health, probiotic products are depicted as both benign CAM and as powerful disease management tools, even within the very same website. This dual messaging may create confusion among patients with chronic GI diseases, who may wonder where these products fit within the context of their current therapies. Of particular concern is that these patients may discontinue medication use without consulting a gastroenterologist, in favor of over-the-counter probiotic products.

The difference in language used to market probiotics to healthy consumers contrasts with marketing strategies designed to elicit interest among patients with chronic GI diseases. In a study by Mercer and colleagues, when GI patients were asked to comment on their personal perceptions of the risks and benefits of probiotics, the authors found that many GI patients were reluctant to initiate new treatments, even over-the-counter treatments, without consulting their physicians [7]. Mercer and colleagues also found that patients expressed concerns that while probiotics may be effective in promoting good digestive health for many individuals, as patients with chronic GI diseases, they felt they needed to exercise greater caution in using products that affect digestive balance. For many of these patients, claims of improved digestive health were not sufficient to motivate probiotic use.

Another source of inconsistent online messaging about probiotics is evident in the frequent suggestion that individuals consult a physician or other medical professional prior to using probiotics. Although the importance of seeking advice from a physician was explicit on many websites this message frequently was accompanied by tips on where to purchase over-the-counter probiotics or invitations to purchase probiotics online. While suggestions to bring physicians into decisions about the use of probiotics may temper patient’s enthusiasm to purchase probiotics independently, appeals to physician involvement may also suggest greater levels of medical benefit than are supported by available data.

We suggest that the responsibility to clarify the potential benefits and risks of probiotic therapies falls squarely in the hands of health care providers. By discussing probiotics with patients in a nonjudgmental manner that is mindful of the many mixed messages that patients may have received concerning these products, HCPs can help patients assess what role, if any, probiotics should play in their ongoing treatment approach.

### Commercial interests

The complex and sometimes inconsistent mix of messages about probiotics presented online are further complicated by a strong presence of commercial interests in many of the websites patients may view. Structurally these sites interface closely with probiotic marketing and frequently identify specific brands or products by name. Even when patients are not offered the opportunity to purchase a particular product online, our data shows that online searches will often include multiple websites promoting the use of probiotic products. The influence of commercial interests is of particular concern since the overall quality of commercial websites, as measured by the Sandvik score, was significantly lower in comparison to websites that lacked a commercial focus. Many commercial websites failed to identify an author, failed to indicate how current the information contained on the site was, and lacked balance. These findings suggest that HCPs should be cautious in promoting any particular product and direct interested patients to more balanced, higher quality websites maintained by noncommercial entities.

In an era when direct-to-consumer advertising plays a major role in the marketing of pharmaceutical drugs [33,34], the strategies employed on probiotic websites are not surprising. In addition, manufacturers’ claims regarding the efficacy of probiotic products are not subjected to the rigorous oversight typical of pharmaceutical drugs [35]. Nonetheless, references to peer-reviewed medical literature appeared in the majority of the websites we reviewed (63%). The presence of such citations implies a certain degree of scientific validity behind claims of safety or efficacy, regardless of the accuracy of the specific statements made or relevance of the studies cited. In a best case scenario, potential inconsistencies between available research data and claims of health benefit would be discussed in a clinical setting, with the involvement of a knowledgeable gastroenterology specialist. However, it is unclear whether patients will seek this additional input from a HCP or pursue probiotics outside clinical settings, as a self-management approach.

### Study limitations

This study of online content related to probiotics has several limitations that reflect our approach to defining an appropriate sample. First and foremost is the challenge of characterizing the ever-changing information available on the Internet. By taking snapshots of relevant websites within a defined time period we were able to minimize the amount of change that may have occurred during data collection and analysis. Subsequent searches of Internet content returned a similar set of websites, suggesting that this information may be reasonably stable. We did not assess the stability of this information formally, however, which is a limitation of our approach. In addition, our analysis of website content, which employed well established qualitative methods and duplicate analysis by two independent coders, has the potential to introduce human error or evaluator bias. Finally, our approach focused narrowly on website content and did not consider how individuals who are exposed to this information may understand or use the information presented. Although not within the scope of our study, our findings highlight the need for additional research examining how patients interpret online information about probiotics and make healthcare choices based on that information.

As ever larger volumes of information are available directly to patients via the Internet, one of the greatest challenges facing HCPs is to stay current with the many health-related messages that patients may encounter outside traditional clinical settings. Although this challenge is not unique to gastroenterology, the marketing and direct availability of probiotic products combine to create additional challenges in maintaining good communication between patients and their HCPs.

Our results suggest that the combination of directive product branding, unsubstantiated claims of health benefit on Internet websites, and direct patient access to probiotics, should be cause for concern among gastroenterologists. The messages patients receive from Internet websites may require that gastroenterologists and other HCPs revisit their patients’ expectations about probiotics and provide a more scientifically grounded and balanced overview of their therapeutic value. These discussions should aim to clarify the potential benefits and risks of probiotics for individuals with chronic GI diseases, highlighting potential differences in the benefit-to-risk profiles of probiotic usage for healthy consumers in comparison to patients with chronic GI diseases. Gastroenterologists may also find it helpful to direct interested patients to well established sources of health information that are free of commercial influence. By initiating these discussions, gastroenterologists and other patient educators can help to establish realistic expectations about probiotics.

## Endnotes

^a^ According to MacMillan Dictionary a content farm is, “A website which publishes huge volumes of low-quality content.”

## Competing interests

The authors declare that they have no competing interests.

## Authors’ contributions

MB contributed to study design, data collection, data analysis and the writing of the manuscript. MBM contributed to study design, data collection, data analysis, and the writing of the manuscript. RS conceived of the study, participated in its design and coordination, and provided critical review and revision of the manuscript.

## Pre-publication history

The pre-publication history for this paper can be accessed here:

http://www.biomedcentral.com/1471-230X/13/5/prepub

## References

[B1] Pew Internet and American Life Project Health topics February 1, 2011 http://www.pewInternet.org/~/media//Files/Reports/2011/PIP_HealthTopics.pdf.

[B2] Boucher J Technology and patient-provider interactions: improving quality of care, but is it improving communication and collaboration? Diabetes Spectrum 2010 23 142 144 10.2337/diaspect.23.3.142

[B3] Cowan M Millennial transformation for primary care Military Med 2010 175 379 381 10.7205/milmed-d-10-00184 20572466

[B4] Murray E Lo B Pollack L The impact of health information on the Internet on the physician-patient relationship Arch Intern Med 2003 163 1727 1734 10.1001/archinte.163.14.1727 12885689

[B5] Hesse B Nelson D Kreps Croyle RT Arora NK Rimer BK Viswanath K Trust and sources of health information: the impact of the Internet and its implications for health care providers: findings from the first health information national trends survey Arch Intern Med 2005 165 2618 2624 10.1001/archinte.165.22.2618 16344419

[B6] O'Connor JB Johanson JF Use of the web for medical information by a gastroenterology clinic population JAMA 2000 284 1962 1964 10.1001/jama.284.15.1962 11035893

[B7] Mercer M Brinich MA Geller G Harrison K Highland J James K Marshall P McCormick JB Tilburt J Achkar JP Farrell RM Sharp RR How patients view probiotics: findings from a multicenter study of patients with inflammatory bowel disease and irritable bowel syndrome J Clin Gastroenterol 2012 46 138 144 10.1097/MCG.0b013e318225f545 21716123 PMC3202682

[B8] Langhorst J Anthonisen IB Steder-Neukamm U Luedtke R Spahn G Patterns of complementary and alternative medicine (CAM) use in patients with inflammatory bowel disease: perceived stress is a potential indicator for CAM use Complement Ther Med 2007 15 30 37 10.1016/j.ctim.2006.03.008 17352969

[B9] Li FX Verhoef MJ Best A Otley A Hilsden RJ Why patients with inflammatory bowel disease use or do not use complementary and alternative medicine: a Canadian national survey Can J Gastroenterol 2005 19 567 573 16151549 10.1155/2005/943547

[B10] Hilsden RJ Verhoef MJ Best A Pocobelli G Complementary and alternative medicine use by Canadian patients with inflammatory bowel disease: results from a national survey Am J Gastroenterol 2003 98 1563 1568 10.1111/j.1572-0241.2003.07519.x 12873578

[B11] Nielsen Wire Top U.S. Online search providers: February 11, 2009 http://blog.nielsen.com/nielsenwire/online_mobile/top-us-online-search-providers-january-2009/.

[B12] Eysenbach G Kohler C How do consumers search for and appraise health information on the world-wide-web? qualitative study using focus groups, usability tests and in-depth interviews BMJ 2002 324 573 577 11884321 10.1136/bmj.324.7337.573 PMC78994

[B13] Sandvik H Health information and interaction on the Internet BMJ 1999 319 29 32 10.1136/bmj.319.7201.29 10390457 PMC28152

[B14] Landis JR Koch GG The measurement of observer agreement for categorical data Biometrics 1977 33 1 159 174 10.2307/2529310 843571

[B15] SPSS for windows [computer program]. version 19.0 2010 SPSS Inc

[B16] Fedorak Richard N Dieleman LA Madsen KL Cohen RD, Wu GU Prebiotics, probiotics, antibiotics, and nutritional therapies in IBD Inflammatory bowel disease, clinical gastroenterology 2011 2 Totowa, NJ: Humana Press 123 150

[B17] Floch MH Walker WA Guandalini S Hibberd P Gorbach S Surawicz C Sanders ME Garcia-Tsao G Quigley E Isolauri E Fedorak RN Dieleman LA Recommendations for probiotic use-2008 J Clin Gastroenterol 2008 42 Suppl 2 104 108 10.1097/MCG.0b013e31816b903f 18542033

[B18] Holubar SD Cima RR Sandborn WJ Pardi DS Treatment and prevention of pouchitis after ileal pouch-anal an astomosis for chronic ulcerative colitis Cochrane Database Syst Rev 2010 6 CD001176 20556748 10.1002/14651858.CD001176.pub2

[B19] Sood A Midha V Makharia GK Ahuja V Singal D Goswami P Tandon RK The probiotic preparation, VSL#3 induces remission in patients with mild to-moderately active ulcerative colitis Clin Gastroenterol Hepatol 2009 7 1202 1209 10.1016/j.cgh.2009.07.016 19631292

[B20] Miele E Pascarella F Giannetti E Quaglietta L Baldassano RN Staiano A Effect of a probiotic preparation (VSL#3) on induction and maintenance of remission in children with ulcerative colitis Am J Gastroenterol 2009 104 437 443 10.1038/ajg.2008.118 19174792

[B21] Mallon P McKay D Kirk S Gardiner K Probiotics for induction of remission in ulcerative colitis Cochrane Database Syst Rev 2007 4 CD005573 17943867 10.1002/14651858.CD005573.pub2

[B22] Hempel S Newberry S Ruelaz A Wang Z Miles JNV Suttorp MJ Johnsen B Shanman R Slusser W Fu N Smith A Roth E Polak J Motala A Perry T Shekelle PG Safety of probiotics to reduce risk and prevent or treat disease. Evidence report/technology assessment No. 200. (Prepared by the southern California evidence-based practice center under contract No. 290-2007-10062-I.) AHRQ publication No. 11-E007 2011 Rockville, MD: Agency for Healthcare Research and Quality http://www.ahrq.gov/clinic/tp/probiotictp.htm.

[B23] Boyle RJ Robins-Browne RM Tang ML Probiotic use in clinical practice: what are the risks Am J Clin Nutr 2006 83 1256 1264 16762934 10.1093/ajcn/83.6.1256

[B24] Zuccotti GV Meneghin F Raimondi C Dilillo D Agostoni C Riva E Giovannini M Probiotics in clinical practice: an overview J Int Med Res 2008 36 Suppl 1 1A 53A 18230282 10.1177/14732300080360S101

[B25] Walker R Buckley M Probiotic microbes: the scientific basis 2006 Washington, DC: American Academy of Microbiology 32687280

[B26] Besselink MG van Santvoort HC Buskens E Boermeester MA van Goor H Timmerman HM Nieuwenhuijs VB Bollen TL van Ramshorst B Witteman B Rosman C Ploeg RJ Brink MA Schaapherder A Dejong C Wahab PJ van Laarhoven CJ van der Harst E van Eijck C Cuesta MA Akkermans L Gooszen HG Probiotic prophylaxis in predicted severe acute pancreatitis: a randomized, double-blind, placebo-controlled trial Lancet 2008 371 651 659 10.1016/S0140-6736(08)60207-X 18279948

[B27] Vogel G Clinical trials: deaths prompt a review of experimental probiotic therapy Science 2008 319 557 18239097 10.1126/science.319.5863.557a

[B28] Mehta DH Gardiner PM Phillips RS McCarthy EP Herbal and dietary supplement disclosure to health care providers by individuals with chronic conditions J Altern Complement Med 2008 14 1263 1269 10.1089/acm.2008.0290 19032071 PMC2787410

[B29] Blendon RJ DesRoches CM Benson JM Brodie M Altman DE Americans’ views on the use and regulation of dietary supplements Arch Intern Med 2001 161 805 810 10.1001/archinte.161.6.805 11268222

[B30] Eisenberg DM Kessler RC Van Rampay MI Kaptchuk TJ Wilkey SA Appel S Davis RB Perceptions about complementary therapies relative to conventional therapies among adults who use both: results from a national survey Ann Intern Med 2001 135 344 351 11529698 10.7326/0003-4819-135-5-200109040-00011

[B31] Adler SR Fosket JR Disclosing complementary and alternative medicine use in the medical encounter: a qualitative study in women with breast cancer J Fam Pract 1999 48 453 458 10386489

[B32] Pappas S Perlman A Complementary and alternative medicine. The importance of doctor-patient communication Med Clin North Am 2002 86 1 10 10.1016/S0025-7125(03)00068-3 11795082

[B33] Liang BA Mackey T Direct-to-consumer advertising with interactive internet media: global regulation and public health issues JAMA 2011 305 824 825 10.1001/jama.2011.203 21343583

[B34] Frosch DL Grande D Tarn DM Kravitz RL A decade of controversy: balancing policy with evidence in the regulation of prescription drug advertising Am J Public Health 2010 100 24 32 10.2105/AJPH.2008.153767 19910354 PMC2791253

[B35] Food and Agriculture Organization of the United Nations and World Health Organization Report on joint FAO/WHO expert consultation on evaluation of health and nutritional properties of probiotics in food including powder milk with live lactic acid bacteria 2001 ftp://ftp.fao.org/es/esn/food/probio_report_en.pdf.

